# Integrating Chemical Footprinting Data into RNA Secondary Structure Prediction

**DOI:** 10.1371/journal.pone.0045160

**Published:** 2012-10-16

**Authors:** Kourosh Zarringhalam, Michelle M. Meyer, Ivan Dotu, Jeffrey H. Chuang, Peter Clote

**Affiliations:** Department of Biology, Boston College, Chestnut Hill, Massachusetts, United States of America; University of North Carolina at Charlotte, United States of America

## Abstract

Chemical and enzymatic footprinting experiments, such as shape (selective 2′-hydroxyl acylation analyzed by primer extension), yield important information about RNA secondary structure. Indeed, since the 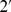-hydroxyl is reactive at flexible (loop) regions, but unreactive at base-paired regions, shape yields quantitative data about which RNA nucleotides are base-paired. Recently, low error rates in secondary structure prediction have been reported for three RNAs of moderate size, by including base stacking pseudo-energy terms derived from shape data into the computation of minimum free energy secondary structure. Here, we describe a novel method, RNAsc (*RNA soft constraints*), which includes pseudo-energy terms for each nucleotide position, rather than only for base stacking positions. We prove that RNAsc is *self-consistent*, in the sense that the nucleotide-specific probabilities of being unpaired in the low energy Boltzmann ensemble always become more closely correlated with the input shape data after application of RNAsc. From this mathematical perspective, the secondary structure predicted by RNAsc should be ‘correct’, in as much as the shape data is ‘correct’. We benchmark RNAsc against the previously mentioned method for eight RNAs, for which both shape data and native structures are known, to find the same accuracy in 7 out of 8 cases, and an improvement of 25% in one case. Furthermore, we present what appears to be the first direct comparison of shape data and in-line probing data, by comparing yeast asp-tRNA shape data from the literature with data from in-line probing experiments we have recently performed. With respect to several criteria, we find that shape data appear to be more robust than in-line probing data, at least in the case of asp-tRNA.

## Introduction

RNA is an important biomolecule, known to play both an *information carrying* and a *catalytic* role. RNA plays roles in numerous biological processes, including *retranslation* of the genetic code (selenocysteine insertion, ribosomal frameshift), transcriptional and translational gene regulation, temperature-dependent allosteric regulation, chemical modification of specific nucleotides in the ribosome, regulation of alternative splicing, apparent regulation of the formation of heterochromatin, etc. (See [1] for a recent review on the analysis of sequence and structure of such noncoding RNA.) Since the function of non-coding RNA largely depends on its structure and since it is believed that RNA plays many yet undiscovered roles in cellular processes, it is important to determine the structure of RNA.

A secondary structure for a given RNA nucleotide sequence 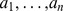 is a set 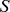 of base pairs 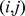, such that 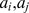 forms either a Watson-Crick or GU (wobble) base pair, and such that there are no *base triples* or *pseudoknots* in 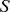. In this context, a base triple in 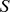 consists of two base pairs 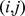, 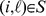 or 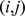, 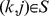. A pseudoknot in 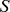 consists of two base pairs 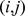, 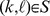 with 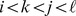. Although it is NP-hard [2] to compute the minimum free energy (MFE) tertiary (or even pseudoknotted) structure of RNA [3], the MFE secondary structure can be computed in time that is cubic in the input sequence length [4]. Moreover, it is widely believed that RNA folds in a hierarchical fashion [5]–[8], with the secondary structure acting as a scaffold for tertiary structure, although this is not universally accepted [9].

RNA secondary structure can be predicted by Zuker and Stiegler's algorithm [4], implemented in mfold [10], RNAfold [11], and RNAstructure [12]. This algorithm uses dynamic programming with free energy parameters from the Turner energy model [13] to compute the minimum free energy (MFE) structure.

A first step towards integrating chemical/enzymatic probing data was taken by Mathews et al. [14], where Zuker and Stiegler's algorithm was modified to support *hard constraints* reflecting the experimental data. In particular, given an RNA sequence, the software RNAstructure [14] computed the minimum free energy (MFE) secondary structure subject to user-defined constraints, such as stipulating that particular nucleotides remain unpaired, that pairs of specific nucleotides form a base pair, etc. Mathews et al. reported that the MFE structure prediction with (hard) constraints corresponding to chemical modification (1-cyclohexyl-3-(2-morpholinoethyl) carbodiimide metho-p-toluene sulfonate, dimethyl sulfate, and kethoxal) yielded an improvement in base-pair accuracy for 5S rRNA of *E. coli* from 26.3% to 86.8% [14]. (See [15] for more remarks and a less optimistic evaluation of RNAstructure with hard constraints on 16S rRNA.)

Chemical/enzymatic probing data is probabilistic in nature, as exemplified in pars footprinting data [16]. Rarely is it absolutely clear that certain positions are unpaired, or that certain base pairs are formed; instead, there is a certain probability of these events. In moving away from error-prone *hard* constraints, Deigan et al. [15] took a second step of incorporating shape (selective 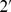-hydroxyl acylation analyzed by primer extension) data [17], [18], whose numerical values (continuously) range from 0 to approximately 2.2, by incorporating a *pseudo free energy* for base stacking into the Zuker algorithm. The pseudo free energy term in [15] was defined to be
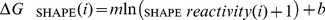
(1)where 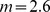 kcal/mol and 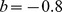 kcal/mol, for each position 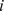 occurring in a base pairing stack; if 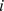 is unpaired, then no pseudo free energy is added. (The position 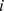 is in a base pairing stack if 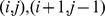 are base pairs, or if 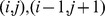 are base pairs belonging to the secondary structure. For base pairs 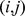 that are surrounded by base pair neighbors 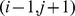 and 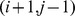, the pseudo-energy term is applied twice.) The resulting modified version of Zuker and Stiegler's algorithm, as implemented in RNAstructure was reported to yield secondary structure prediction accuracies of up to 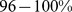 for three moderate-sized RNAs (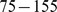 nt) and for 16S rRNA (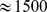 nt). Wilkinson et al. [19] later described a model for the secondary structure of the HIV-1 genome, as computed by RNAstructure with shape pseudo energies defined in equation 1. If correct, this is a remarkable feat, given that the size of the HIV-1 genome is generally just under 10,000 nt (see http://www.hiv.lanl.gov), hence several times larger than the ribosome, whose crystal structure was only determined after years of painstaking work (the large unit, PDB code 1FFK [20], of the ribosome of *Haloarcula marismortui* consists of a 23S chain of length 2,922 nt and a 5S chain of 122 nt).

One issue with this approach is that it takes into consideration shape data only for base-stacked positions, i.e., a pseudo free energy term corresponding to shape data is applied at positions where a stacked base pair occurs, but not where nucleotides are unpaired. By ignoring shape data for unpaired nucleotide positions, this approach can thus bias structure prediction to form base pairs even at positions, which shape data may suggest are flexible. Indeed the expected distance of predicted base pairing probabilities computed by RNAstructure with shape values increases after the incorporation of the shape pseudo energy terms (see Table 1). (As later defined, RNAstructure and RNAsc both compute the probability 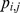 that base pair 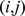 belongs to a structure in the low energy Boltzmann ensemble. Since the pseudo energy model for shape data incorporation is different in RNAstructure and RNAsc, the base pairing probabilities and Boltzmann low energy ensembles may be different.) In contrast to the pseudo energies of RNAstructure, our algorithm RNAsc, will always shift the distribution of conformations towards the shape measurements (see Methods for a mathematical proof).

**Table 1:** Benchmark results.

Secondary structure prediction accuracy											
RNA	len	test	(A)	(B)	(C)	RNA	len	test	(A)	(B)	(C)
asp-tRNA	75	sens.	1.00	1.00	0.76	phe-tRNA	76	sens.	1.00	0.75	0.95
		ppv	1.00	1.00	0.76			ppv	0.95	0.71	0.95
		ave ent.	0.21	0.17	0.27			ave ent.	0.2	0.17	0.46
		str. div.	19.53	17.17	22.60			str. div.	11.37	9.38	34.37
		edist.	23.7	61.77	24.9			edist.	29.51	61.77	33.68
HCV IRES	95	sens.	0.96	0.96	0.96	5S rRNA	120	sens.	0.94	0.94	0.26
		ppv	1.00	1.00	1.00			ppv	0.82	0.82	0.22
		ave ent.	0.05	0.06	0.27			ave ent.	0.30	0.17	0.27
		str. div.	3.20	3.57	21.45			str. div.	46.93	20.70	32.90
		edist.	31.36	52.48	36.53			edist.	42.57	54.01	46.41
P546	155	sens.	0.95	0.96	0.43	glycine	162	sens.	0.92	0.92	0.70
		ppv	0.96	0.98	0.44			ppv	0.84	0.84	0.61
		ave ent.	0.18	0.12	0.38			ave ent.	0.11	0.05	0.30
		str. div.	27.7	14.05	66.50			str. div.	15.14	5.13	44.16
		edist.	41.36	131.77	56.11			edist.	53.90	115.55	60.29

A comparison of three secondary structure prediction algorithms, using shape data from Deigan et al. [15] for the three RNA molecules, yeast aspartyl tRNA (asp-tRNA), hepatitis C virus internal ribosomal entry site (HCV IRES), and the P546 domain from the bI3 group I intron (P546), along with shape data from [26] for three additional RNA molecules, *E. coli* phenylalanine tRNA (phe-tRNA), *E. coli* 5S ribosomal RNA (5S rRNA), and *F. nucleatum* glycine riboswitch (glycine). The benchmark results are tabulated for (A) RNAsc+shape, (B) RNAstructure+shape, and (C) RNAstructure (with no shape data). Sensitivity 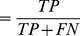 is abbreviated by sens., positive predictive value 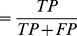 is abbreviated by ppv. The average pointwise entropy, Morgan-Higgs structural diversity, and the expected distance of the computed probabilities to the probing data are abreviated by ave ent., str. div., and edist., respectively. Not shown: results for medloop and *V. vulnificus* adenine riboswitch (1Y26), for which all three methods have optima sensitivity and ppv values of 1.0.

Nonetheless, MFE dynamic programming methods that incorporate high throughput chemical/enzymatic footprinting data can yield important insights into the structure and function of RNA molecules, much faster than the labor-intensive X-ray diffraction methods.

The motivation for our work is to develop a method that incorporates chemical/enzymatic footprinting data in a *self-consistent* manner. In particular, given experimental data of the form 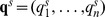, where 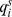 is the experimental probability that the 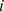
th nucleotide is *unpaired* (or, more accurately, in a flexible region, as witnessed by high shape reactivity), our goal is to develop an algorithm incorporating footprinting data such that the *recalculated* probabilities 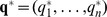 are guaranteed to be closer to the experimental measurements. If our algorithm is self-consistent in this manner, then we have strong mathematical evidence that the partition function computation and hence the MFE computation are both as correct as is the shape data. In contrast to the pseudo energies of RNAstructure, we prove that our algorithm RNAsc is self-consistent, and on average, the ensemble of low energy secondary structures produced by our method yields a footprinting pattern that closely resembles the pattern from input experimental shape data. We benchmark our method against the RNAstructure program [19] on eight RNAs, for which shape data and native structures are both available. The secondary structure predictions from our method and from RNAstructure are fairly similar and both significantly improve secondary structure prediction without incorporation of footprinting data (e.g. mfold, RNAfold). However, the expected distance of the computed probabilities with the shape data is lower in our method for all the test cases. It is worth noting that the mistakes in the predicted secondary structure usually occur in positions where the shape data might be inaccurate, or where the native structure and shape data structures could be somewhat different, due to quite different temperatures required by each experimental protocol. Recent studies have shown that different experimental mapping approaches can provide complementary structural information [21]. Thus, we additionally performed in-line probing [22], [23] on asp-tRNA, in order to compare the results of shape and in-line probing in the context of our algorithm. The source code of RNAsc as well as a web server is available at http://bioinformatics.bc.edu/clotelab/RNAsc/.

## Methods

### In-line probing experiments

**Figure 1:**
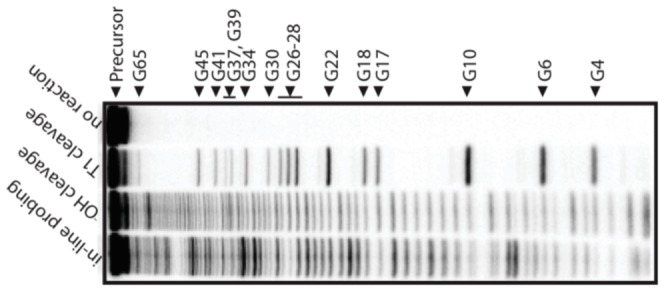
In-line probing. Spontaneous cleavage pattern resulting from in-line probing of yeast asp-tRNA, nucleotides with larger backbone flexibility will have higher rates of cleavage and thus bands of greater intensity. Lanes for no reaction, T1 RNase (cleavage following only guanosines), and partial hydroxyl cleavage (-OH, cleavage after each base) are indicated. Due to the high resolution of the gel, double bands appear for nucleotides 2–9. These bands correspond to RNA molecules where the 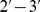 cyclic phosphate intermediate has hydrolyzed to leave either no phosphate, or a mixture of 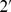
- and 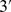-phosphate products which migrate more quickly on the gel. Quantifcation of these positions combined the bands corresponding to both products. The precursor RNA and T1 RNase cleavage products are marked. Not all guanosines show cleavage due to retention of secondary structure at 5 M urea and elevated temperature.

DNA oligonucleotides for the sequence and its reverse complement were purchased from MWG Operon; remaining reagents were obtained from Sigma-Aldrich. DNA oligonucleotides were annealed to create templates for T7 polymerase transcription, and the transcription products were purified by denaturing PAGE and eluted in 10 mM Tris-HCl (pH 7.5 at 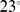
C), 200 mM NaCl and 1 mM EDTA. Following in-line probing protocols designed by the Breaker Lab [22], [23], synthesized RNA molecules were dephosphorylated using alkaline phosphatase (Roche Diagnostics) and radiolabeled with [g-32P]ATP and T4 polynucleotide kinase (NEB) according to the manufacturers instructions. Spontaneous transesterification reactions using PAGE-purified, 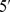 endlabeled RNAs were assembled as described in [23]. Incubations were performed for approximately 40 h at 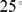
C in 10-uL volumes containing 50 mM Tris-HCl (pH 8.3 at 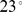
C), 20 mM MgCl2, 100 mM KCl and 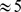 nM RNA. RNA fragments resulting from spontaneous transesterification were resolved by denaturing 10% PAGE, and imaged with a Molecular Dynamics STORM PhosphorImager. Quantification of gels were performed using SAFA (Semi-Automated Footprinting Analysis) [24]. In-line probing experiments were repeated an additional two times, resulting in gels with comparable data (data not shown). Fig. 1 is an image of the in-line probing gel for yeast asp-tRNA.

### Computational methods

Briefly stated, our algorithm, RNAsc (*RNA soft constraints*), consists of a preprocessing step, that normalizes shape data to the range 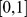, followed by a computation of the minimum free energy [resp. partition function], which incorporates pseudo-energy terms [resp. Boltzmann factors of pseudo-energy terms] for each nucleotide position. We begin by discussion of the normalization of shape data.

#### Normalization of shape

**Figure 2:**
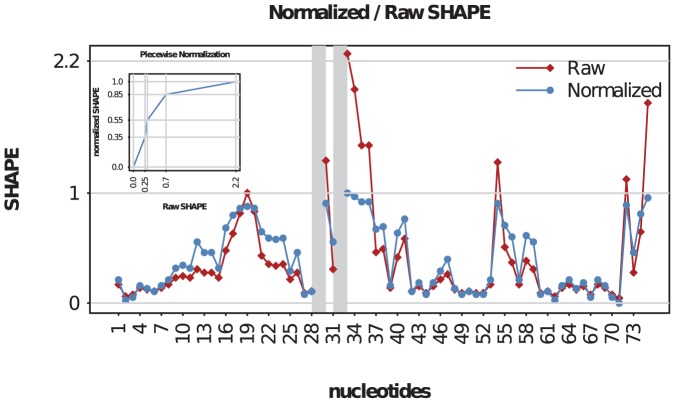
Normalization. Normalized (blue circles) and raw (red diamonds) shape values. Gray bars indicate the missing shape values. The subplots shows the piecewise normalization map.

In experiments reported by the Weeks Lab [25] as well as the Das Lab [26], shape reactivities range from 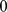 to roughly 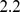. Large reactivities suggest that the position is unpaired; small reactivities suggest that the position is base-paired. More specifically, nucleotides with shape reactivities 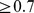 or 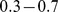 are considered highly and moderately reactive, respectively [15]. The normalization is carried out in a piecewise linear fashion where 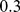 will be roughly mapped to 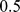. However, very low shape reactivities should not be mapped close to 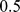 either as it will bias the shape values toward unpaired nucleotides. For this reason the shape reactivity values 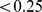 are linearly mapped to the interval 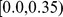, the reactivity values in 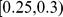 are linearly mapped to the interval 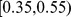, the reactivity values in 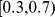 are linearly mapped to the interval 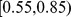, and lastly, the reactivities 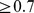 are linearly mapped to the interval 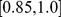. The selection of the threshold values are motivated by the moderate and high reactivity thresholds as reported in [15] and the examination of the cumulative distribution of the shape data (see File S1). The in-line probing data was normalized by mapping the outliers at the 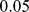 and the 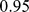 quantiles to 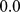 and 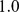 respectively and normalizing the rest of the data to 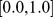 linearly. Fig. 2 shows a plot of the normalized and raw shape values as well as the normalization map.

#### Boltzmann weights

Let 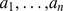 be a fixed RNA sequence of length 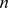, for which we are given normalized shape or in-line probing reactivity data 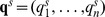, where 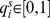. For 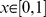 and 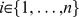, define the Boltzmann weight
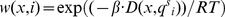
(2)where 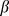 is a scaling parameter, and 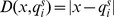 measures the discrepancy between 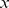 and 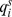. We will later incorporate Boltzmann weights in a *weighted* partition function 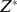, in a manner that reweights the ensemble of low energy conformations towards the shape data. When later used in recurrence relations for 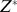, the variable 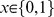 is the indicator function for whether a position is unpaired 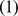 or paired 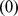 in a secondary structure under consideration. In the case of missing values, 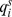 may be assigned to 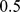, which represents no information about base pairing.

#### Weighting the partition function

In this section, we describe how to integrate Boltzmann weights into the computation of the partition function for secondary structures of a given RNA sequence.This allows us to compute the probability 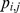 [resp. 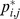] that 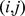 is a base pair in the Boltzmann ensemble of structures, where weights for shape or in-line probing have not [resp. have] been taken into consideration. As later explained, we will compare the probability 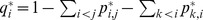 with normalized shape reactivity 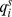. Let 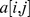 denote the subsequence 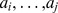 of a given, fixed RNA sequence 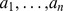 of length 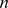. For 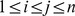, the McCaskill [27] partition function 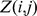 is defined by 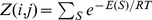, where the sum is taken over all secondary structures 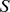 of 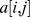, 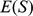 is the free energy of 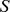 with respect to the Turner energy model [13], [28], 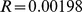 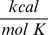 is the universal gas constant, and 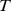 absolute temperature. The goal of the current paper is to integrate the previously defined weights into the partition function. We first require some notation. Here, we write 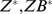, etc. instead of the more cumbersome notation 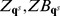, etc. Thus 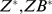 etc. depend on the normalized footprinting data 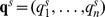, although 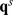 will not be explicitly mentioned.

#### Definition 1 (Weighted partition function)

*Define*

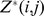
: weighted partition function over all secondary structures of 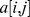.

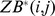
: weighted partition function over all secondary structures of 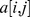, which contain the base pair 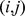.

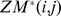
: weighted partition function over all secondary structures of 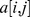, subject to the constraint that 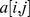 is part of a multiloop and has *at least* one component.

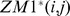
: weighted partition function over all secondary structures of 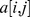, subject to the constraint that 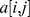 is part of a multiloop and has *exactly* one component. Moreover, it is *required* that 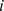 base-pair in the interval 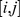; i.e. 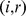 is a base pair, for some 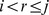.

To compute partition function 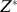, we compute by dynamic programming 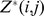 for all 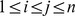 by increasing values of 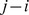. Structures on 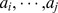 can be subdivided into those for which 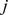 is unpaired in 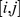, thus contributing 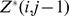 times Boltzmann factor for 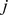 to be unpaired, and those for which 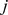 is paired with 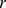 for 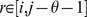, thus contributing 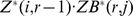 times Boltzmann factor for 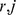 to be paired. Subsequently 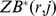 is computed by adding a contribution for all loops closed by base pair 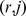, i.e., hairpins, bulges, internal loops and multi loops whose latter contribution is recursively computed by jultiloop partition functions 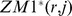 and 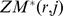. In essence, we apply Boltzmann weights to each nucleotide position 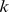, while accounting for a distinct weight depending on whether 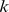 is paired or unpaired in the structure 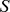 under consideration: weight 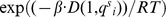 if 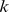 is unpaired in 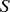, weight 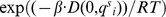 if 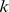 is base-paired in 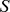. If all weights were set to 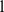, then the weighted partition function would be equivalent to the classic partition function. Similar forms of rearranging and reweighting of the partition function have been applied in the context of single stranded RNA binding proteins [29]. Details now follow. It will be expedient to define the function 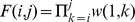, which represents the weight corresponding to a *loop* region in which 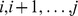 are unpaired. For 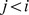, 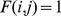, while for 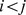,
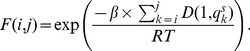
(3)In the base case, we define 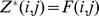 and 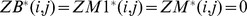 for all 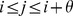, where 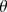 is the minimum number of unpaired bases in a hairpin loop (generally 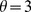). In the inductive case, where 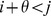, we define
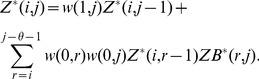
(4)Note that in the above equation 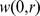 and 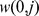 correspond to the weights for the nucleotides 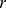 and 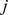 being paired, but not necessarily to one another. If extra information on the pairing status of the nucleotides is available, (e.g., as in ‘mutate and map’ experiments [30]), these weights may be corrected accordingly to reflect the weight for the pairing of the 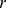
th and the 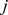
th nucleotides. Let 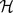 denote the free energy of a hairpin and let 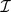 denote the free energy of an internal loop (which combines the cases of stacked base pair, bulge and proper internal loop). The free energy for a multiloop containing 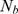 base pairs and 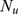 unpaired bases is given by the affine approximation 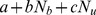. The weighted partition function closed by base pair 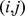 is given by
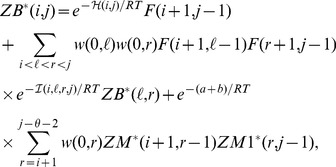
(5)The weighted multiloop partition function with a single component and where position 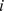 is required to base-pair in the interval 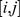 is given by
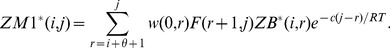
(6)Finally, the weighted multiloop partition function with one or more components, having no requirement that position 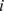 base-pair in the interval 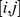 is given by
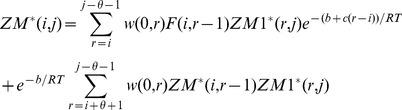
(7)The weighted Boltzmann probability of base pair 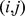 is defined by
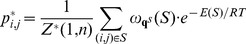
(8)where 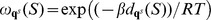 – see Methods. Following Zuker [31], the inner and outer partition function is computed, from which we easily obtain 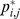.

The minimum free energy (MFE) structure can be computed by a modification of McCaskill's algorithm [27], where the weighted partition function is modified by replacing summations by minimizations, products by sums, and replacing the weights by 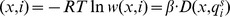. Although we did implement this algorithm, it does not include energy contributions for stacked, single-stranded nucleotides (dangles) or coaxial stacking, both known to be important in improving secondary structure prediction accuracy. For this reason, we modified the source code of RNAstructure, for both the MFE as well as the partition function computation which implements dangles and coaxial stacking. See File S1 for details. As in [15], the value of the scaling parameter 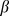, is determined by a search to optimize positive predictive value and sensitivity.

#### Measures of uncertainty in the predicted low-energy ensemble of conformations

Pointwise entropy and Morgan-Higgs structural diversity [32] were used as measures of uncertainty in the prediction of the secondary structure. The poinwise entropy is defined as follows. For each *fixed* 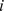 in 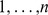, define probability distribution 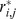 on 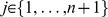 by setting 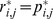 for 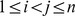, 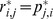 for 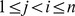, and 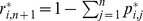. Pointwise entropy 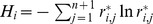 measures the *variability* in nucleotides found to be base-paired with 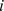 in the Boltzmann ensemble of low energy structures. The pointwise entropy without the probing data is computed similarly using the probabilities 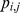. To reflect the nature of the probing data, we modified this definition as follows. Define the binary pointwise entropy at position 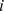 by 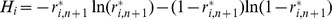. Binary entropy measures the uncertainty in the 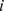
th nucleotide being paired or unpaired, reflecting the signal detected by probing data. Similar computations were done with 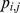 (the base pairing probabilities without the integration of the weights). The Morgan-Higgs structural diversity is defined by 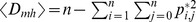, where 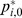 is defined by 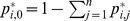. Similar computations were done with 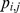.

### RNAsc is guaranteed to improve agreement with shape data

In this section, we show that on average, the ensemble of low energy secondary structures produced by our method yields a footprinting pattern that more closely resembles the pattern from input experimental shape data; in particular, we prove that the expected distance from (normalized) shape data for the ensemble of low energy structures (our algorithm) is strictly less than the expected distance from shape data for the Boltzmann ensemble of low energy structures (McCaskill's algorithm). First, we require some definitions. All secondary structures 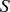 considered in this section will be tacitly assumed to be secondary structures of the RNA molecule 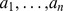. Each secondary structure 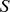 can be assigned a binary sequence 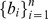 so that 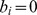 if the nucleotide 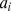 is paired and 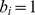 otherwise. Given experimental shape data yielding probabilities 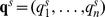, where 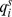 is the probability that nucleotide 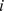 is *unpaired*, the distance of 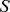 to 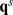 is defined by:
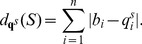
(9)The shape *weight* of 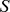 is defined to be
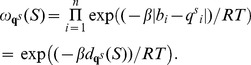
(10)The weighted partition function then becomes
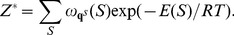
(11)The *Boltzmann probability* 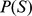 of secondary structure 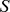 is defined by
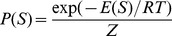
(12)and the *weighted Boltzmann probabity* 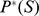 is defined by
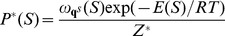
(13)Define the *critical distance* 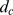 by
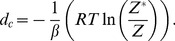
(14)Note that 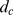 does not depend on any particular secondary structure 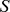, although it does depend on 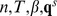 and of course the input RNA sequence 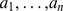. It follows from definitions that for any secondary structure 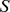,
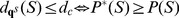
(15)and strict inequalities hold as well. Indeed, since the exponential function is increasing, we have 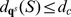 if and only if
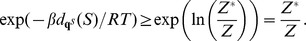
Multiplying each side by 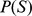, the above inequality can be written as
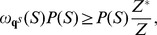
from which (15) follows. Similarly,
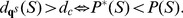
(16)


Next, define the expected distance 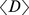 between 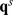, obtained by normalizing shape data, and the ensemble of low energy structures as follows:
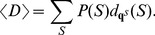
(17)Similarly, define the SHAPE *weighted expected distance* 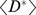 between 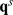 and the ensemble of low energy structures by
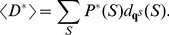
(18)Let 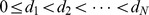 represent the sorted distances 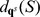 between all secondary structures of 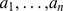, for given normalized shape data 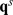. Here 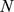 denotes the total number of secondary structures. Note that there may be many distinct secondary structures that have a given distance 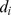 to 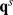; i.e. possibly many distinct 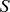 for which 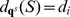. Let 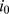 be the largest index 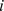 such that 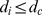; it follows that 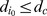 and 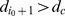. Let 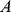 [resp. 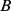] consist of those secondary structures 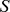, such that 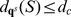 [resp. 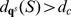]; in other words
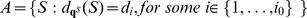


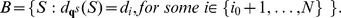



Theorem 1: For any given RNA sequence 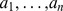 and normalized SHAPE data 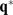, 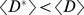.

Proof:
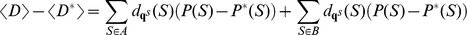


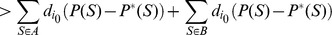


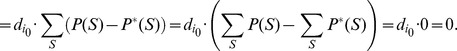
To justify the inequality, note that for 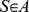, 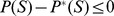, hence for 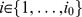, we have 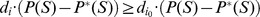. On the other hand, for 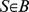, 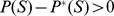, hence for 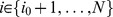, we also have 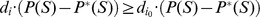. Finally, the last line follows from the fact that 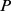 and 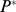 are both probability distributions, hence 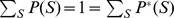. This completes the proof that 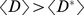.

The above theorem can be generalized; however, we first require some notation. The weighted partition function 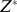, weighted Boltzmann probability 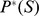, and weighted expected distance 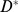 were respectively defined in Equations (11),(13), and (18). When we wish to make the weighting parameter 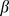 explicit, we instead write 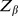, 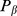 and 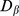. The following theorem shows that as the parameter 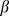 increases, the expected distance to normalized shape data decreases:

Theorem 2: For any given RNA sequence 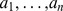, normalized SHAPE data 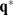 and 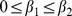, 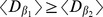; moreover, strict inequalities hold as well.

The proof the the theorem can be found in File S1.

### Quadratic time computation of expected distance from shape data

Given RNAsc parameter 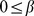, recall that we defined the 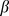-expected distance 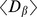 between 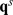, obtained by normalizing shape data, and the ensemble of low energy structures by
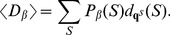
(19)In the main text, we wrote 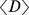, instead of 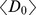 when 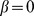.

In trying to compute 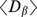 by definition, we seemingly require the sum over exponentially many secondary structures, or at least to approximate this sum by summing over a reprentative sample of structures, sampled from the low energy ensemble. This is not necessary. Here, we show how to compute 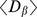 from the base pairing probabilities 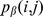, thus leading to a quadratic time algorithm.

By definition,
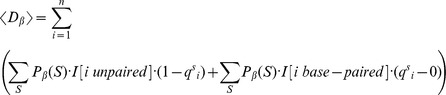
where 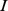 is denotes the indicator function. Now for any fixed 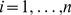,
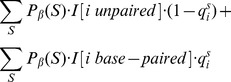
is equal to
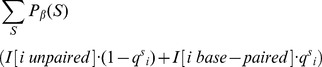
(20)Since 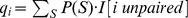, it follows that Equation (20) is equal to
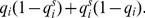
(21)It follows that
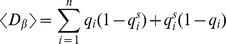
The values 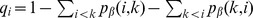 are computed in quadratic time from McCaskill's algorithm, and subsequently stored in an array. If follows that 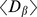 can be computed in quadratic time.

Since RNAstructure of Deigan et al. [15] takes unnormalized shape data in the range from 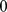 to 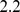, we define the expected distance 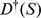 between *unnormalized* shape data and structure 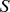 to be
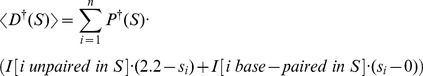
(22)where 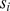 denotes the unnormalized shape data at position 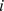. The expected distance 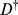 between *unnormalized* shape data and the ensemble of low energy structures computed by RNAstructure with incorporated shape data by
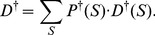
(23)Scrutiny of the proof just given yields an efficient computation of
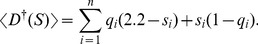
(24)


Since the approach in [15] only considers stacked base pairs, it seems very likely that 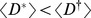, where 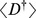 denotes the expected distance from shape data for the Boltzmann ensemble of low energy structures after the incorporation of the shape pseudo energy terms as in [15]. Indeed, the expected distance we obtain between *u*nnormalized input shape data 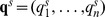 and the computed probabilities 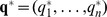 demonstrates this fact (see Table 1).

## Results

In this section we present the benchmarking results for our algorithm RNAsc, a novel algorithm that recalibrates probing data as probabilities of nucleotides being unpaired and integrates this information as ‘soft constraints’ into the computation of minimum free energy secondary structure (see Methods). Furthermore, we present a direct comparison of in-line probing data and shape data for yeast asp-tRNA.

### Analysis of shape and in-line probing for structure prediction

**Figure 3:**
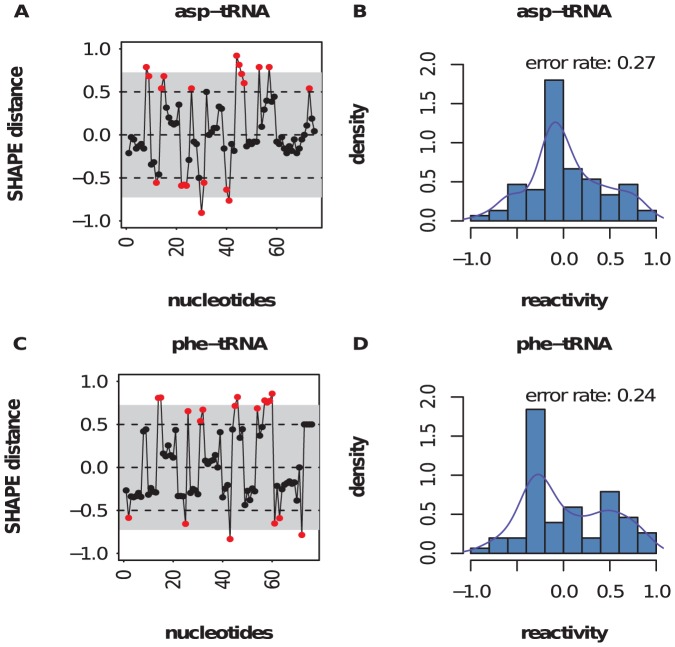
Shape discrepancies. Distribution of shape discrepancies in yeast asp-tRNA *(top)* and *E. coli* phe-tRNA *(bottom)*. shape data for asp-tRNA [resp. phe-tRNA] from the Weeks Lab [25] [resp. Das Lab [26]]. Using crystal structure as ‘gold standard’, red squares indicate locations where the absolute value of the difference of shape data and crystal structure (1 unpaired, 0 paired) exceeds 0.5. The plots on the right show the distribution of the discrepancy in shape as well as the error rate.

In order to directly characterize how well shape data reflects RNA secondary structure, we compared normalized shape data with base pairing status, as determined from crystallographic or NMR structures. We define shape *distance* to equal the difference between *normalized* shape reactivity (see Methods), scaled from 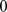 to 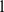 (see section Normalization of shape) and binary base pairing status, with 0 for paired, 1 for unpaired, as derived from NMR or crystal structure. Using shape data for *S. cerevisiae* apartyl-tRNA [25], HCV IRES [15], bI3 group I intron p456 [33], *E. coli* phenylalanine-tRNA [26], *E. coli* 5S RNA [26], and *Fusobacterium nucleatum* glycine riboswitch [26], we computed shape distance at each nucleotide. We observed that at many positions the shape distance has an absolute value greater than 0.5, thus indicating a significant difference between shape reactivity and the actual secondary structure. We refer to these positions as discrepancies. Over the the set of RNAs we examined, between 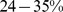 of the total data corresponded to such discrepancies (Fig. 3 and File S1). Many factors can account for these discrepancies, including differences between the crystal structure and the ensemble of structures in solution, potential tertiary contacts, and differential reactivity to the chemical agent.

To assess whether an alternative experimental method might yield data that more accurately reflects the secondary structure, we performed in-line probing on the *S. cereviseae* aspartyl-tRNA, for which shape data is available [25]. Like shape, in-line probing is a measure of backbone flexibility, where nucleotides in loops and other unpaired regions are generally more reactive than those that are base-paired [34]. In-line probing takes advantage of the spontaneous transesterification reactions responsible for RNA degradation that occur only when the 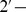
O from one nucleotide and the 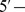
O of the next align in a 180 degree conformation around the phosphate. This conformation does not occur in the A-form helix, thus protecting linkages within the helix from cleavage. In-line probing and shape are thus likely to yield similar, but not equivalent data [35].

**Figure 4:**
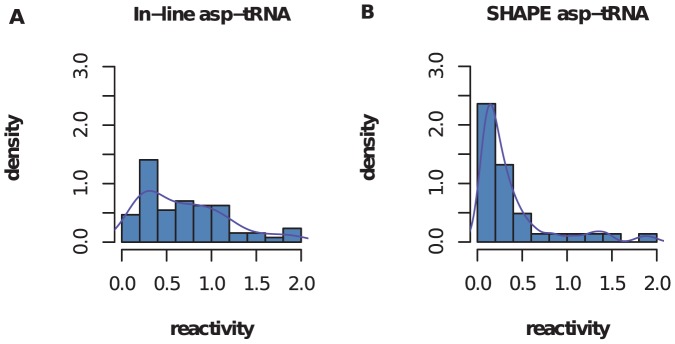
Comparison of In-line probing and shape. Distribution of reactivities of data from in-line probing *(A)* and shape *(B)*. In-line probing reactivities were determined using SAFA [24] and then normalized to range 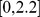, in order to be comparable with shape reactivities. Histograms suggest that in-line probing signal is more diffuse than that from shape. The fraction of base-pairs in asp-tRNA is 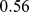 which could be used to estimate the threshold shape moderate reactivity.

Our analysis indicates that in-line probing and shape reactivity profiles are quite distinct from one another. See Fig. 4 for a comparison of shape and in-line probing profiles and File S1 for shape reactivity profiles of other RNA molecules.

The signal from in-line probing is significantly more diffuse than that from shape, and the error rate, as calculated above for shape, is significantly higher (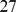 vs. 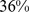). Thus shape is a better reflection of secondary structure than in-line probing, at least in the case of asp-tRNA.

**Figure 5:**
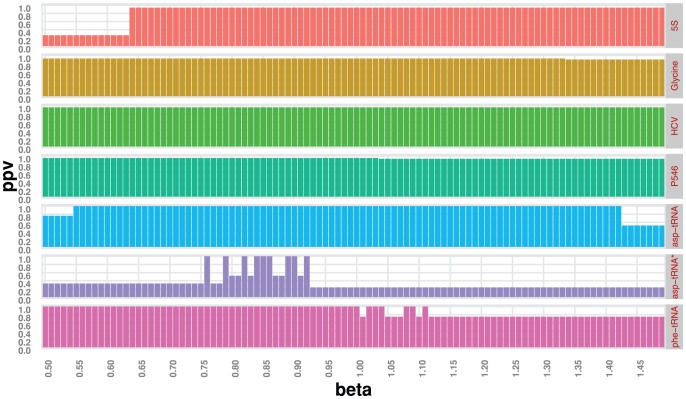
Optimal parameter value. The plots show heat maps displaying ppv (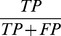) as a function of parameter 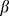 for RNAsc with data from shape and in-line probing *(asp-tRNA* 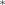 *)*. Note the much larger area for good parameter choices when using shape data, rather than in-line probing data. This data suggests that shape data is more robust than in-line probing data, when used in computing MFE structure with RNAsc. Computations were done at 37

**Figure 6:**
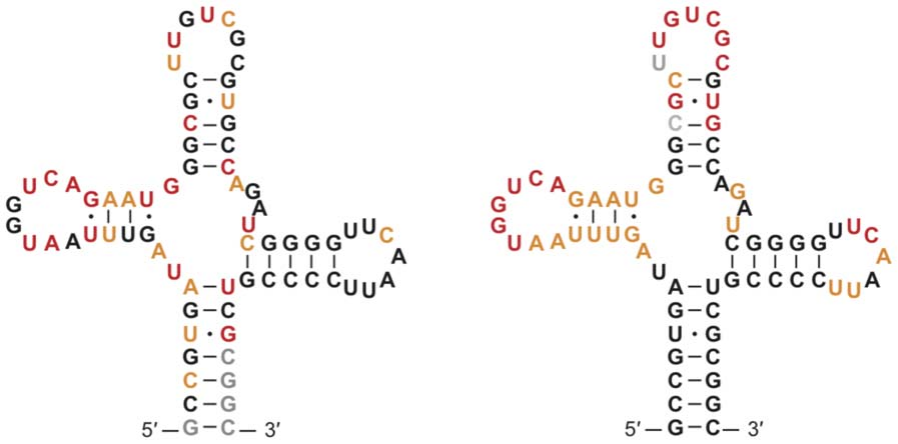
Heat maps of in-line probing and shape. Heat maps illustrating differences between in-line probing *(left)* and shape *(right)* analysis of the yeast asp-tRNA. Nucleotides are colored corresponding to cumulative activities described in Figure 3, where the least reactive 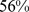 of bases are black (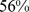 of bases are paired in the crystal structure), the most reactive 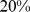 of bases are red, and the next most reactive 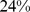 are yellow. Gray bases are bases for which there is no data available.

Integrating shape and in-line probing data into our new algorithm RNAsc also shows that shape has an edge over in-line probing. The structures predicted by RNAsc for yeast asp-tRNA using in-line probing and shape data are both identical to the crystal structure. However, one measure of the robustness of the data in the context of our secondary structure prediction algorithm RNAsc is the range of the scaling parameter 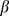 over which the correct structure can be recovered. Recall that 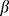 is a weight parameter (see section Boltzmann Weights for details). We conducted a search for parameter 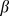 for yeast asp-tRNA, using both in-line probing data and shape data. We found that when using in-line probing data, RNAsc produced the target structure for asp-tRNA only for a very narrow range of 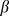, while when using shape data, this range was much larger (see Fig. 5). See Fig. 6 for a heat map of in-line vs. shape reactivity for asp-tRNA.

C.

**Figure 7:**
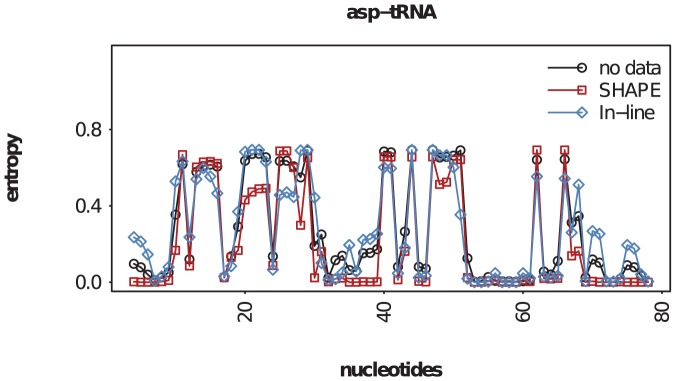
Pointwise entropies. Pointwise entropy of yeast asp-tRNA, computed from RNAsc using shape data (red squares), in-line probing (blue diamonds), and using no probing data (black circles). Average pointwise entropies: 0.210 (shape data), 0.267 (in-line probing), 0.269 (no data). As expected, by integrating either shape or in-line probing data into RNAsc, the variability (entropy) decreases; however, it appears that variability (entropy) is decreased more by shape than by in-line probing data – again, suggesting that shape data is more robust than in-line probing data when used with RNAsc.

In a second analysis, we compared the pointwise entropy at each nucleotide using no data, shape data, and in-line probing data (see Fig. 7). We observe that shape data decreases the average entropy more than in-line probing data. However, we also observe that there are positions where the in-line probing decreases the entropy more than shape, suggesting that combinations of different experimental approaches may be able to yield additional information.

### Validation of RNAsc

Using shape data from the Weeks Lab, we tested RNAsc on aspartyl-tRNA from *S. cerevisiae*, domain II of the hepatitis C virus internal ribosomal entry site (HCV IRES), and the P546 domain of the bI3 group I intron, from *E. coli*. Additionally, using shape data from the Das Lab, we tested RNAsc on *E. coli* phenylalanine tRNA (phe-tRNA), *E. coli* 5S ribosomal RNA (5S rRNA), and the glycine riboswitch from *F. nucleatum* with PDB code 3P49. As ‘gold standard’ structures, we used NMR structure for P546, and X-ray structures for remaining RNAs. Parameter used for RNAsc is 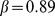, determined by search (see Fig. 5) to optimize sensitivity (proportion of true positives that are correctly identified) and positive predictive value (proportion of positive results that are true positives). Slippage of 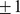 [15], [36] is *not* allowed, contrary to benchmarking results of some authors. Here, slippage [36] means that if base pair 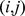 is in the true structure, then the base pair 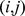 is counted as “correctly” predicted, if one of the base pairs 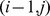, 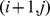, 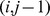, 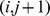 appears in the predicted structure – we do *not* allow slippage in the results of this paper.

Table 1 presents a comparison of RNAsc with RNAstructure, including a comparison of structural variation in the ensemble of low energy structures. This variation is computed by pointwise entropy and Morgan-Higgs structural diversity (see Methods). The table shows that the low energy ensemble, as computed by RNAsc with integration of shape data, has intermediate variation between that computed by RNAstructure with and without shape data. The fact that RNAstructure with incorporated shape data computes an ensemble of structures with less variation appears to be expected, given the parameters used in the algorithm of Deigan et al. [15].

As explained in Deigan et al. [15], RNAstructure incorporates shape data by including a pseudo free energy term
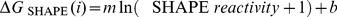
(25)for a nucleotide position 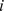. In the source code RNAstructure, it is clear that the pseudo free energy term 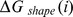 is applied *only* for positions 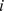 involved in a stacked base pair. The optimal values for slope 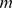 and 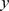-intercept 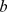 are obtained by grid search when maximizing structure prediction accuracy on certain known structures. Optimal slope and intercept values reported in [15] are 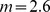 and 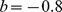 kcal/mol.

We now show that the smaller structural variation in the RNAstructure ensemble appears to be an artifact of the magnitude of parameters 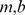. Consider the two most extreme cases: (1) position 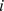 in structure 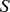 is base-paired, but shape reactivity is a maximum, (2) position 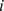 in structure 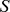 is not paired, but shape reactivity is a minimum.

Suppose that position 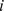 is in a base-stacked region but the shape reactivity at position 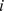 is 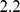, a maximum, though there are sometimes shape reactivities larger than 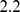. With the default parameters for 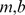, the pseudo free energy contribution of RNAstructure is 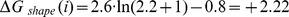, an energetic penalty. This penalty is quite large, given the fact that the largest (in absolute value) free energy contribution for base stacking is 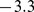 kcal/mol [37]. Under the same assumptions, RNAsc would have a pseudo free energy of 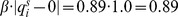, also an energetic penalty, yet much smaller than that of RNAstructure.

Suppose now that position 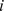 is in a loop region but the shape reactivity at position 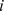 is 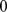, the least possible value. Using the default parameters 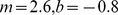 kcal/mol, the pseudo free energy contribution of RNAstructure, *if* applied in this case, would then be 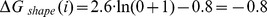. This value, paradoxically, would be an energetic bonus, although the predicted structure disagrees with shape data! It is presumably for this reason that Deigan et al. do not apply any pseudo free energy term to nucleotide positions 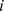 located in a loop region. In contrast, under the same assumptions, RNAsc would have a pseudo free energy of 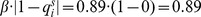, again a penalty – moreover, the same penalty of 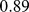 kcal/mol is applied in each of the cases (1) and (2) just discussed.

From these illustrative examples, it is suggestive that structural *variability*, as measured by pointwise entropy and structural diversity, in the low energy ensemble calculated by RNAstructure is *higher* than that of the RNAsc low energy ensemble, due to the magnitude of the parameters 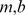 used in RNAsc.

**Figure 8:**
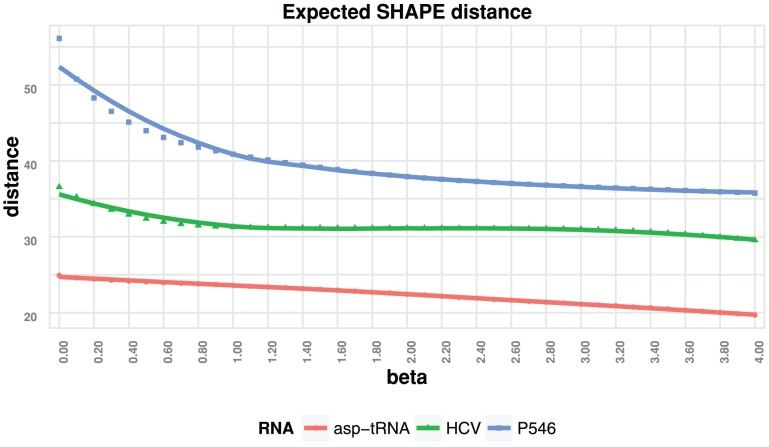
Expected distance of predicted probabilities with normalized shape data. The figure shows a plot of the expected distance 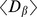 between normalized experimental shape values 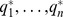 and the low energy Boltzmann ensemble, as computed by RNAsc. The 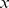-axis depicts increasing values of RNAsc parameter 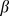, while the 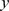-axis depicts expected distance 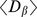. The curves confirm the statement of Theorem 2, which states that as 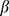 increases, the expected distance 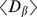 decreases. The figure also shows that for higher values of 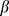, 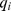 can be made to agree very closely 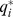. The expected distances of the predicted probabilities with *u*nnormalized shape values for RNAstructure are 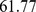, 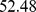, and 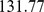 for asp-tRNA, HCV, and P546 respectively using optimal parameter values (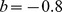 and 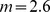).

Note that the average relative decrease in expected distance of the computed probabilities to shape data from RNAstructure to RNAsc is 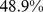. In fact the expected distance of the computed probabilities to shape *increases* for RNAstructure and *decreases* for RNAsc after the incorporation of shape in each case. Apart from the ‘self-consistent’ nature of our algorithm, not shared by RNAstructure, the demonstrable expected distance of the computed probabilities to shape data provided by our approach, indicates that we account more fully for the shape data. It is worth mentioning that for higher values of 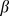 the predicted Boltzmann probabilities 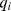 can be made to agree very closely with the experimental values 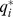 (strong self-consistency). Fig. 8 shows a plot of the expected distance of the computed probabilities to shape data for increasing values of 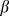 – see Methods for a proof. Note however that since the experimental probabilities (or normalized shape values) are generally not in perfect agreement with the native structure, we took the closeness of the predicted structure to the native structure as a measure for choosing the parameter 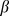.

We believe RNAsc may be helpful long-term in elucidating the nature of discrepancies between shape and the native structure. As in any experimental protocol, there is a Gaussian error term; however, our data (not shown) indicates that shape discrepancy is positively correlated with high pointwise entropy. Indeed, it seems plausible that a region of the RNA molecule which fluctuates due to thermal motion, thus having higher pointwise entropy, might entail a more variable accessibility for the chemical probe NMIA, thus causing a greater shape discrepancy with the X-ray structure. The program RNAsc allows the user to determine such regions of high pointwise entropy, and to see the structure variability in that region by sampling. It may be possible to confirm or refute our hypothesis concerning the non-Gaussian nature of shape discrepancy (“error”), by performing additional shape probing experiments at lower temperatures. It follows that RNAsc could prove to be a valuable tool in this line of research.

## Discussion

**Figure 9:**
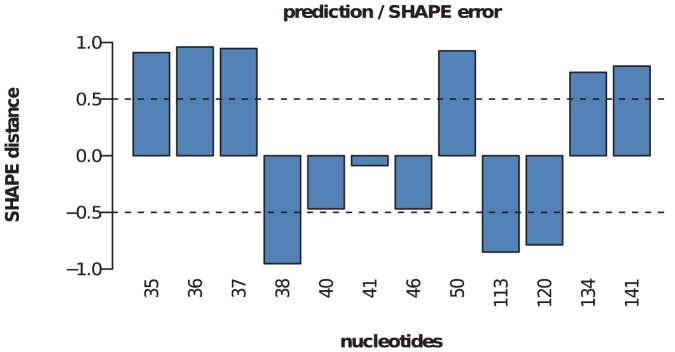
Errors in the prediction of the secondary structure of glycine riboswitch by RNAsc. On the 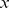-axis, nucleotide positions are displayed, where the algorithm predicts the structure incorrectly. The 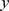-axis represents the shape distance to the native structure at the given nucleotide. A shape distance with absolute value 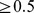 indicates an error.

Widespread accessibility of quantitative RNA structural mapping techniques and medium- to high-throughput quantification of the data have motivated the development of computational tools to predict structures from such information. The integration of experimental data as “constraints” in the thermodynamic algorithm when computing minimum free energy (MFE) structure can significantly improve the accuracy of RNA structure prediction. However, such methods are also dependent on the quality of the data used for the constraints [26]. It is worth mentioning that the errors in our algorithm RNAsc are directly related to the errors in the experimental data. Fig. 9 shows shape distance to the native structure at the nucleotides where the secondary structure is predicted incorrectly for glycine riboswitch. As can be seen, the shape distances to the native structure are very large for 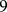 out of the 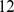 incorrectly predicted positions. Thus the prediction errors are due to the quality of the input data rather than limitations of the algorithm.

Two recent approaches towards overcoming this error include the iterative ‘sample and select’ approach of Quarrier et al. [38] and the ‘mutate and map’ strategy of Kladwang et al. [30]. The ‘sample and select’ strategy involves multiple mapping, followed by a simple filtering step, which removes the suboptimal structures (sampled from the low energy ensemble using the Sfold software [39]) that are incompatible with mapping data. In contrast, the ‘mutate and map’ strategy involves high-throughput structural probing of all single-nucleotide mutants, resulting in 2D shape data, followed by a computation of the minimum free energy structure, in which pseudo-energy base stacking terms have been added that correspond to Z-scores from 2D shape data. Although high-throughput ‘mutate and map’ strategies [30], using either shape -CE (capillary electrophoresis) or shape -Seq [40], provide very high secondary structure prediction accuracy, such methods also represent a significant increase in both experimental manipulation and cost that is often not warranted for more specific studies. Especially in such cases, we believe that our method, RNAsc, may be the tool of choice. On the other hand, the ‘mutate and map’ strategy can be normalized in such a way as to obtain base pairing probabilities. Since shape experiments can potentially probe tertiary interactions (as mentioned in the previous section), not only could we obtain probabilities for secondary interactions and canonical base pairs, but also for tertiary and long range interactions as well as non-canonical base pairs. These probabilities can later be used as input to algorithms such as Probknot [41] or even to a Maximum Weight Matching algorithm [42] to predict pseudoknotted structures and non-canonical base pairs. We are currently pursuing this line of research.

## Supporting Information

File S1 Supplementary information.
(PDF)
