# Supplementary material for: Integrating Chemical Footprinting Data into RNA Secondary Structure Prediction
Source: PLoS One. 2012 Oct 16;7(10):e45160. doi: 10.1371/journal.pone.0045160 (PMC3473038; doi:10.1371/journal.pone.0045160)
Supplement: File S1 — Supplementary information. (PDF) [file pone.0045160.s001.pdf]

Supporting Information: Integrating chemical footprinting data into RNA secondary structure prediction

Kourosh Zarringhalam[1,†], Michelle M. Meyer[1], Ivan Dotu[1], Jeffrey H. Chuang[1‡], and Peter Clote[1,*]
1 Department of Biology, Boston College, Chestnut Hill, MA
∗ E-mail: peter.clote@bc.edu
† Present address: Department of Mathematics, University of Massachusetts Boston, Boston, MA
‡ Present address: The Jackson Laboratory for Genomic Medicine, Farmington, Connecticut

Increasing β decreases average distance to normalized SHAPE data

In this section, we generalize the theorem from the text to show that as `RNAsc` parameter β increases, the expected distance to normalized SHAPE data decreases. The proof, which generalizes the proof of the theorem in the main text, is given below.

First, we begin with some notation. Thoughout this section, S denotes a secondary structure of an arbitary, but fixed RNA sequence $a_1, \ldots, a_n$. As in the text, each secondary structure S is associated with a binary sequence $b_1, \ldots, b_n$ such that $b_i = 1$ if the nucleotide a_i is unpaired and $b_i = 0$ if a_i is base-paired. Given experimental SHAPE data yielding probabilities $\mathbf{q}^s = (q_1^s, \ldots, q_n^s)$, where q_i^s is the probability that nucleotide i is *unpaired*, the distance of S to $\mathbf{q}^s$ is defined by:

$$d_{\mathbf{q}^s}(S) = \sum_{i=1}^{n} |b_i - q_i^s|. \tag{1}$$

For secondary structure S and parameter $\beta \geq 0$ in the algorithm `RNAsc`, SHAPE *weight* for (S, β) is defined to be

$$\begin{aligned} \omega_{\mathbf{q}^s}(S,\beta) &= \prod_{i=1}^{n} \exp\left((-\beta|b_i - q_i^s|)/RT\right) \\ &= \exp\left((-\beta d_{\mathbf{q}^s}(S))/RT\right). \end{aligned} \tag{2}$$

The β-weighted partition function then becomes

$$Z_\beta = \sum_{S} \omega_{\mathbf{q}^s}(S,\beta) \exp(-E(S)/RT). \tag{3}$$

The *β-weighted Boltzmann probability* $P(S)$ of secondary structure S is defined by

$$P_\beta(S) = \frac{\omega_{\mathbf{q}^s}(S,\beta)\exp(-E(S)/RT)}{Z_\beta} \tag{4}$$

Let $0 \leq \beta_1 \leq \beta_2$ be arbitrary, but fixed values for the parameter β in `RNAsc`. Define the *critical distance* $d_c(\beta_1, \beta_2)$ by

$$d_c(\beta_1,\beta_2) = \frac{-RT \ln\left(\frac{Z_{\beta_2}}{Z_{\beta_1}}\right)}{\beta_2 - \beta_1}. \tag{5}$$

Note that $d_c(\beta_1, \beta_2)$, which has the form of a change in ensemble free energy, does not depend on any particular secondary structure S, although it does depend on $n, T, \beta_1, \beta_2, \mathbf{q}^s$ and of course the input RNA sequence $a_1, \ldots, a_n$.

CLAIM 1: If $d_1 \leq d_2$ and $\beta \geq 0$, then

$$\exp(-\beta d_1/RT) \geq \exp(-\beta d_2/RT).$$

Claim 1 is obvious. We now prove the following principal claim.

Claim 2: For any secondary structure S,

$$d_{\mathbf{q}^s}(S) \le d_c(\beta_1, \beta_2) \iff P_{\beta_1}(S) \ge P_{\beta_2}(S) \tag{6}$$

and strict inequalities hold as well.

For notational ease, let d_c abbreviate $d_c(\beta_1, \beta_2)$. By Equation (5) and Claim 1, for any S,

$$d_{\mathbf{q}^s}(S) \le d_c \iff d_{\mathbf{q}^s}(S) \le \frac{-RT \ln(Z_{\beta_2}/Z_{\beta_1})}{\beta_2 - \beta_1},$$

hence

$$\exp\left(\frac{-(\beta_2 - \beta_1) d_{\mathbf{q}^s}(S)}{RT}\right) \ge \exp\left(\frac{-(\beta_2 - \beta_1) d_c}{RT}\right) = \frac{Z_{\beta_2}}{Z_{\beta_1}}.$$

Multiply both sides of the last line by $P(S) = \frac{\exp(-E(S)/RT)}{Z}$ to obtain

$$P_{\beta_2}(S) = P(S) \cdot \frac{\exp\left(\frac{-\beta_2 d_{\mathbf{q}^s}(S)}{RT}\right)}{Z_{\beta_2}} \ge P(S) \cdot \frac{\exp\left(\frac{-\beta_1 d_{\mathbf{q}^s}(S)}{RT}\right)}{Z_{\beta_1}} = P_{\beta_1}(S).$$

This establishes Claim 2.

For $0 \le \beta$, define the β-expected distance $\langle D_\beta \rangle$ between $\mathbf{q}^s$, obtained by normalizing SHAPE data, and the ensemble of low energy structures by

$$\langle D_\beta \rangle = \sum_S P_\beta(S) d_{\mathbf{q}^s}(S). \tag{7}$$

When $\beta = 0$, we write $\langle D \rangle$, instead of $\langle D_0 \rangle$.

Define disjoint sets A, B of secondary structures S of $a_1, \ldots, a_n$ by

$$\begin{aligned} A &= \{S : d_{\mathbf{q}^s}(S) \le d_c(\beta_1, \beta_2)\} \\ B &= \{S : d_{\mathbf{q}^s}(S) > d_c(\beta_1, \beta_2)\}. \end{aligned}$$

It follows by definition that for all $S \in A$, $d_{\mathbf{q}^s}(S) \le d_c$, and for all $S \in B$, $d_{\mathbf{q}^s}(S) > d_c$.

Theorem: For any given RNA sequence $a_1, \ldots, a_n$, normalized SHAPE data $\mathbf{q}^*$ and $0 \le \beta_1 \le \beta_2$, $\langle D_{\beta_1} \rangle \ge \langle D_{\beta_2} \rangle$; moreover, strict inequalities hold as well.

Proof:

$$\begin{aligned} \langle D_{\beta_1} \rangle - \langle D_{\beta_2} \rangle &= \sum_{S \in A} d_{\mathbf{q}^s}(S) \left(P_{\beta_1}(S) - P_{\beta_2}(S)\right) + \sum_{S \in B} d_{\mathbf{q}^s}(S) \left(P_{\beta_1}(S) - P_{\beta_2}(S)\right) \\ &> \sum_{S \in A} d_c \left(P_{\beta_1}(S) - P_{\beta_2}(S)\right) + \sum_{S \in B} d_c \left(P_{\beta_1}(S) - P_{\beta_2}(S)\right) \\ &= d_c \cdot \sum_S \left(P_{\beta_1}(S) - P_{\beta_2}(S)\right) = d_c \cdot \left(\sum_S P_{\beta_1}(S) - \sum_S P_{\beta_2}(S) \right) = d_c \cdot 0 = 0. \end{aligned}$$

To justify the inequality, note that for $S \in A$, $P_{\beta_1}(S) - P_{\beta_2}(S) \le 0$, hence for $S \in A$, we have $d_{\mathbf{q}^s}(S) \cdot (P_{\beta_1}(S) - P_{\beta_2}(S)) \ge d_c \cdot (P_{\beta_1}(S) - P_{\beta_2}(S))$. On the other hand, for $S \in B$, $P_{\beta_1}(S) - P_{\beta_2}(S) > 0$, hence for $S \in B$, we also have $d_{\mathbf{q}^s}(S) \cdot (P_{\beta_1}(S) - P_{\beta_2}(S)) \ge d_c \cdot (P_{\beta_1}(S) - P_{\beta_2}(S))$. Finally, the last line follows from the fact that P_{β_1} and P_{β_2} are both probability distributions, hence $\sum_S P_{\beta_1}(S) = 1 = \sum_S P_{\beta_2}(S)$. This completes the proof that $\langle D_{\beta_1} \rangle \ge \langle D_{\beta_2} \rangle$. The proof for inequality is similar.

SHAPE discrepancies

In order to directly characterize how well SHAPE data reflects RNA secondary structure, we compared normalized SHAPE data with base pairing status, as determined from crystallographic or NMR structures. We define SHAPE *distance* to equal the difference between *normalized* SHAPE reactivity (see Methods), scaled from 0 to 1, as just defined, and binary base-pairing status, with 0 for paired, 1 for unpaired, as derived from NMR or crystal structure. Using SHAPE data for *S. cerevisiae* apartyl-tRNA [1], HCV IRES [2], bI3 group I intron p456 [3], *E. coli* phenylalanine-tRNA [4], *E. coli* 5S RNA [4], and *Fusobacterium nucleatum* glycine riboswitch [4], we computed SHAPE distance at each nucleotide. We observed that at many positions the SHAPE distance has an absolute value greater than 0.5, thus indicating a significant difference between SHAPE reactivity and the actual secondary structure. We refer to these positions as discrepancies. Over the the set of RNAs we examined, between $24-35\%$ of the total data corresponded to such discrepancies (see Fig. 1).

Cumulative distributions and Histograms of SHAPE reactivity

Nucleotides with SHAPE reactivities ≥ 0.7 or $0.3-0.7$ are considered highly and moderately reactive, respectively [2]. Hence it is reasonable to normalize the SHAPE reactivities in a piecewise linear fashion, where 0.3 will be roughly mapped to 0.5. However, very low SHAPE reactivities should not be mapped close to 0.5 either. For this reason the SHAPE reactivity values < 0.25 are linearly mapped to the interval [0.0.35), the reactivity values in $[0.25, 0.3)$ are linearly mapped to the interval $[0.35, 0.55)$, the reactivity values in $[0.3, 0.7)$ are linearly mapped to the interval $[0.55, 0.85)$, and lastly, the reactivities ≥ 0.7 are linearly mapped to the interval $[0.85, 1.0]$. See Fig. 2 for the distribution of SHAPE reactivities.

Integrating pseudo-energy terms into the Partition function recursions

In this section, we provide the full recursive definitions necessary to compute the partition function, where pseudo-energy factors have been included for every nucleotide position. The recursions are adaptations of recursions from [5]. Corresponding recursions for the minimum free energy (MFE) secondary structure are obtained by replacing the Boltzmann factors of energy by the energy, replacing addition by minimization, and replacing multiplication by addition– hence will not be given explicitly.

Definition 1 *Define:*

- $V(i,j)$*: The partition function for the fragment from nucleotides* i *to* j*, inclusive, with* i *paired to* j*.*
- $W(i,j)$*: The partition functions for the fragment from nucleotides* i *to* j*, inclusive, such that this fragment will be incorporated in a multibranch loop and it has one single helical branch.*
- $WL(i,j)$*: The partition functions for the fragment from nucleotides* i *to* j*, inclusive, such that this fragment will be incorporated in a multibranch loop and it has one single helical branch. Also* j *is required to terminate the helical branch as either a paired nucleotide, a* $3'$ *dangling end, or a nucleotide in a terminal mismatch.*
- $WMB(i,j)$*: The partition function from nucleotides* i *to* j*, inclusive, such that this fragment will be incorporated into a multibranch loop and it contains two or more branches.*

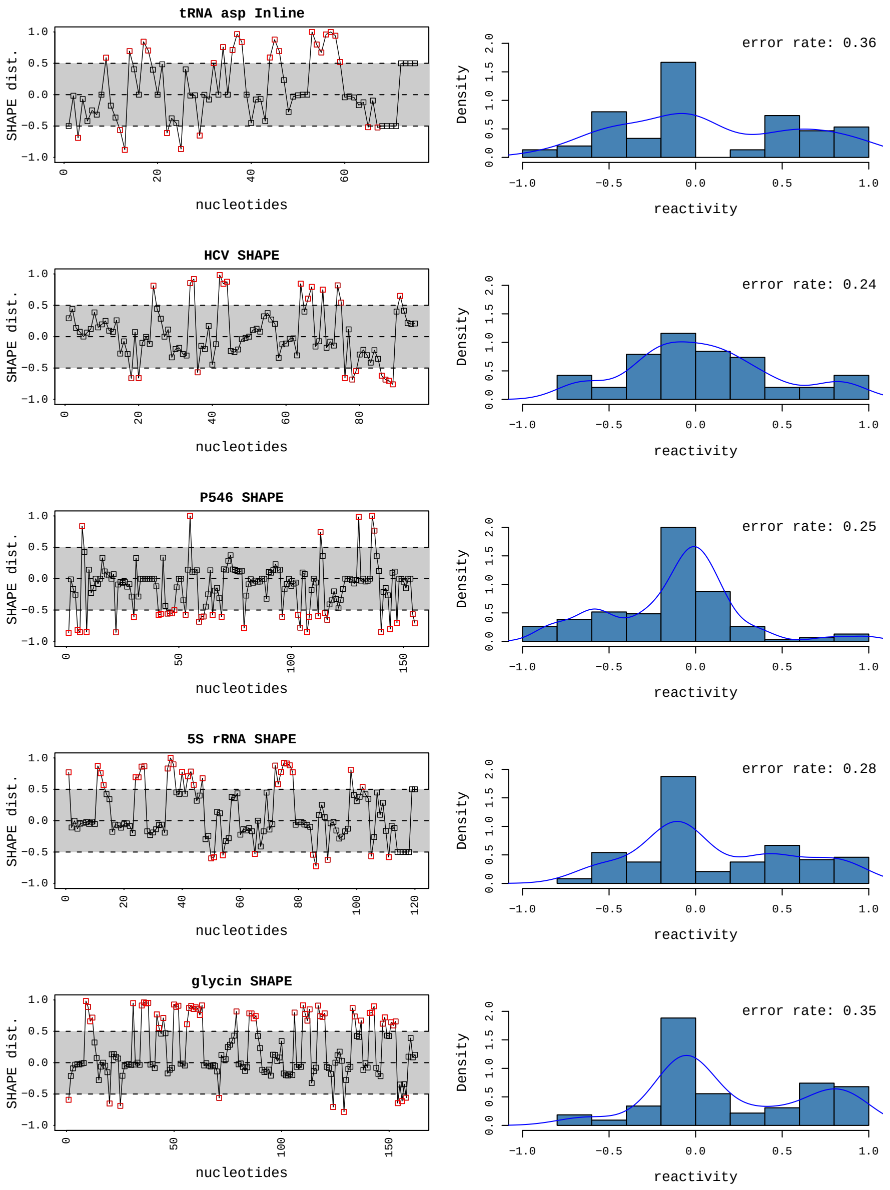

Figure 1. **SHAPE discrepancies.** Distribution of SHAPE discrepancies for east tRNA-Asp, HCV IRES [2], bI3 group I intron p456 [3], *E. coli* 5S RNA [4], and *Fusobacterium nucleatum* glycine riboswitch [4].Using crystal structure as 'gold standard', red squares indicate locations, where the absolute value of the difference of SHAPE data and crystal structure (1 unpaired, 0 paired) exceeds 0.5.

- $WMBL(i,j)$: *The partition function from nucleotides* i *to* j*, inclusive, such that this fragment will be incorporated into a multibranch loop and it contains two or more branches. Also* j *is required to be paired or associated with a helix as either a* $3'$ *dangling end, a nucleotide in a terminal mismatch, or a nucleotide in a mismatch between two coaxially stacked helices.*
- $WC(i,j)$: *The partition function from nucleotides from* i *to* j*, inclusive, such that there are two*

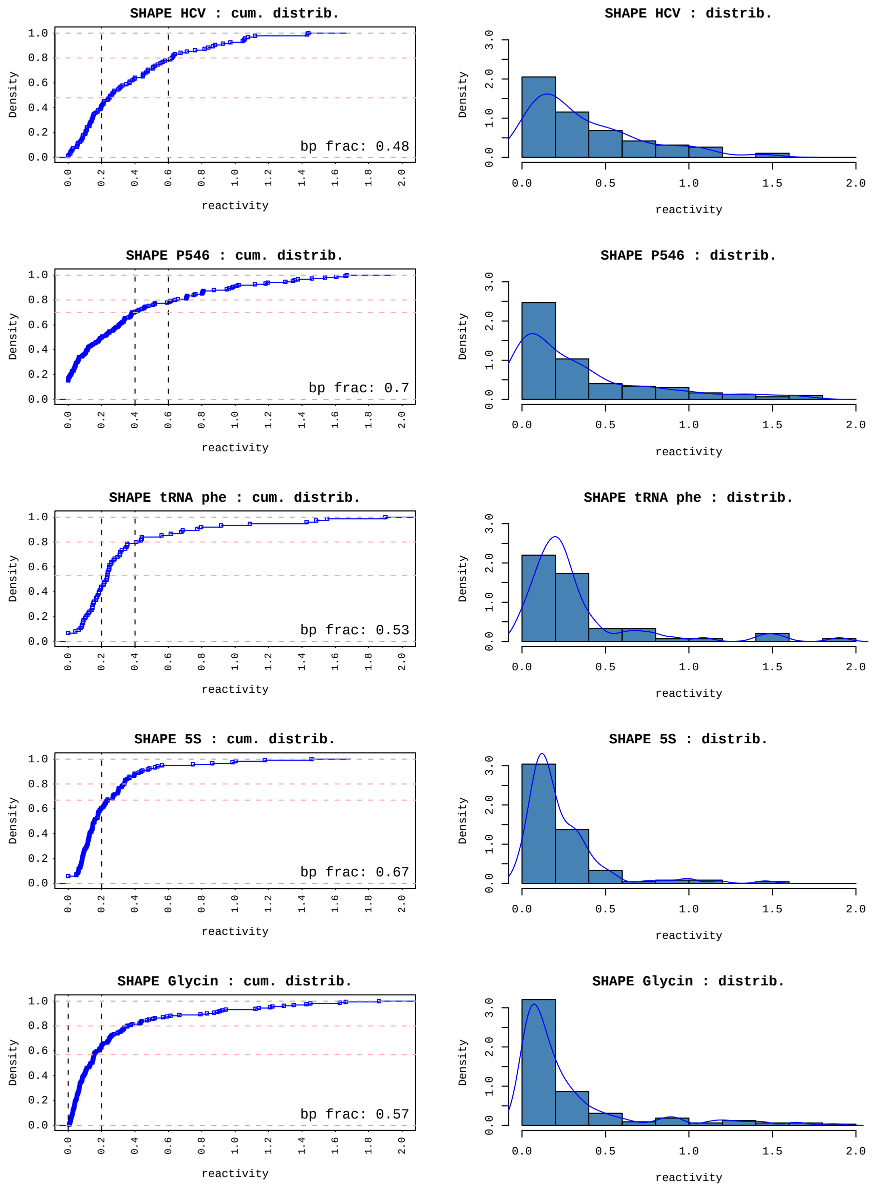

Figure 2. SHAPE **reactivity distributions.** Distribution of reactivities of data for HCV IRES [2], bI3 group I intron p456 [3], *E. coli* phenylalanine-tRNA [4], *E. coli* 5S RNA [4], and *Fusobacterium nucleatum* glycine riboswitch [4].The fraction of base-pairs could be used to estimate the threshold SHAPE moderate reactivity.

coaxially stacked branches. Nucleotides i and j must either be paired, e.g. (i,k), $(k+1,j)$, or be in a single mismatch separating the two helices, e.g. $(i+1,k-1)$, $(k+1,j)$ or (i,k), $(k+2,j-1)$.

- $W5(i)$: *The partition function for the nucleotide fragment from the $5'$ end of the sequence to and including nucleotide i*

- $W3(i)$: *The partition function from and including nucleotide i to the* $3'$ *end of the sequence.*

See [5, 6] for details on the storage and calculation of the arrays. V is the sum of terms involving a pair between i and j, base pair stacking, hairpin loop closure, internal loop closure, and multibranch loop closure. For $j \leq N$, we have:

$$V(i,j) = \{Vh(i,j) + Vs(i,j) + Vi(i,j) + Vm(i,j)\} \times \omega(0,i) \times \omega(0,j),$$

and for $j > N$ we have:

$$V(i,j) = \{Vs(i,j) + Vi(i,j) + Vm(i,j) + Ve(i,j)\} \times \omega(0,i) \times \omega(0,j).$$

Note that V is not defined for $i = N$ and for $j = N + 1$ (i.e., the ends of the sequence.) Vh is the energy contribution of the hairpin and is defined by

$$Vh(i,j) = \begin{cases} e^{-\Delta G(\text{hairpin})/RT} \times F(i+1, j-1) & \text{for } j \leq N, \\ 0 & \text{else.} \end{cases}$$

Vs is the stacking contribution (of a base pair on a previous pair) and is defined by:

$$Vs(i,j) = e^{-\Delta G(stack)/RT} \times V(i+1, j-1).$$

Vi is the energy contribution of internal loops which also considers bulge loops. Vi is defined by,

$$Vi(i,j) = \sum_{i',j'} V(i',j')F(i+1, i'-1)F(j'+1, j-1) \times e^{-\Delta G(stack)/RT}.$$

Computation of Vi requires a search over i' and j' where $i < i' < j' < j$ except where $i = i + 1$ and $j = j - 1$ simultaneously, that is, the base pair stacking case. The search is limited to internal loops of size 30 nt that is, $i' - i + j - j' - 2 \leq 30$, to limit the algorithm to $O(N^3)$. In what follows $3'$ d refers to $3'$ *dangles*, $5'$ d refers to $5'$ *dangles*, TM refers to *Terminal mismatch* and CS refers to *Coaxial Stacking.* Vm is the multibranch contribution and is define by

$$\begin{aligned} Vm(i,j) = {} & WMB(i+1, j-1) \times e^{-(a+c)/RT} + e^{-\Delta G(3'\text{ d})/RT} \\ & \times WMB(i+2, j-1) \times e^{-(a+b+c)/RT} \times \omega(1, i+1) + e^{-\Delta G(5'\text{ d})/RT} \\ & \times WMB(i+1, j-2) \times e^{-(a+b+c)/RT} \times \omega(1, j-1) + e^{-\Delta G(\text{TM})/RT} \\ & \times WMB(i+2, j-2) \times e^{-(a+2b+c)/RT} \times \omega(1, i+1) \times \omega(1, j-1) \\ & + \sum_k e^{-\Delta G(\text{CS})/RT} \times V(i+1, k) \times \{W(k+1, j-1) + WMB(k+1, j-1)\} \times e^{-(a+2c)/RT} \\ & + \sum_k e^{-\Delta G(\text{CS})/RT} \times V(k, j-1) \times \{W(i+1, k-1) + WMB(i+1, k-1)\} \times e^{-(a+2c)/RT} \\ & + \sum_k e^{-\Delta G(\text{CS})/RT} \times V(i+2, k) \times \{W(k+2, j-1) + WMB(k+2, j-1)\} \times e^{-(a+2b+2c)/RT} \\ & \times \omega(1, i+1) \times \omega(1, k+1) + \sum_k e^{-\Delta G(\text{CS})/RT} \times V(k, j-2) \\ & \times \{W(i+1, k-2) + WMB(i+1, k-2)\} \times e^{-(a+2b+2c)/RT} \times \omega(1, k-1) \times \omega(1, j-1). \end{aligned}$$

Ve is the energy contribution from the exterior loops. It is defined only when $j > N$:

$$\begin{aligned}
Ve(i,j) &= W3(i+1) \times W5(j-1-N) + W3(i+2) \times W5(j-1-N) \times e^{-\Delta G(3'\text{ d})/RT} \\
&\times \omega(1,i+1) + W3(i+1) \times W5(j-2-N) \times e^{-\Delta G(5'\text{ d})/RT} \times \omega(1,j-1-N) \\
&+ W3(i+2) \times W5(j-2-N) \times e^{-\Delta G(\text{TM})/RT} \times \omega(1,i+1) \times \omega(1,j-1-N) \\
&+ \sum_k e^{-\Delta G(\text{CS})/RT} \times V(i+1,k) \times W3(k+1) \times W5(j-1-N) \\
&+ \sum_k e^{-\Delta G(\text{CS})/RT} \times V(k,j-1-N) \times W3(i+1) \times W5(k-1) \\
&+ \sum_k e^{-\Delta G(\text{CS})/RT} \times V(i+2,k-2) \times W3(k) \times W5(j-1-N) \\
&\times \omega(1,i+1) \times \omega(1,k-1) + \sum_k e^{-\Delta G(\text{CS})/RT} \times V(i+2,k) \times W3(k+1) \\
&\times W5(j-2-N) \times \omega(1,i+1) \times \omega(1,j-1-N) + \sum_k e^{-\Delta G(\text{CS})/RT} \\
&\times V(k+1,j-2-N) \times W3(i+1) \times W5(k-1) \times \omega(1,j-1-N) \times \omega(1,k) \\
&+ \sum_k e^{-\Delta G(\text{CS})/RT} \times V(k,j-2-N) \times W3(i+2) \times W5(k-1) \\
&\times \omega(1,j-1-N) \times \omega(1,i+1).
\end{aligned}$$

$W5(i)$ is the fragment from 1 to i and is computed by:

$$\begin{aligned}
W5(i) &= W5(i-1) \times \omega(1,i) + \sum_k W5(k) \times V(k+1,i) + \sum_k W5(k) \times e^{-\Delta G(3'\text{ d})/RT} \\
&\times V(k+1,i-1) \times \omega(1,i) + \sum_k W5(k) \times e^{-\Delta G(5'\text{ d})/RT} \times V(k+2,i) \\
&\times \omega(1,k+1) + \sum_k W5(k) \times e^{-\Delta G(\text{TM})/RT} \times V(k+2,i-1) \\
&\times \omega(1,k+1) \times \omega(1,i) + \sum_{k,m} W5(k) \times e^{-\Delta G(\text{CS})/RT} \times V(k+1,m) \\
&\times V(m+1,i) + \sum_{k,m} W5(k) \times e^{-\Delta G(\text{CS})/RT} \times V(k+1,m) \times V(m+2,i-1) \\
&\times \omega(1,m+1) \times \omega(1,i) + \sum_{k,m} W5(k) \times e^{-\Delta G(\text{CS})/RT} \times V(k+2,m-1) \\
&\times V(m+1,i) \times \omega(1,k+1) \times \omega(1,m).
\end{aligned}$$

$W5(0)$ is initialized to 1.

$W3$ is defined similarly, as follows.

$$\begin{aligned}W3(i) = {} & W3(i+1) \times \omega(1,i) + \sum_k W3(k) \times V(i,k-1) + \sum_k W3(k) \times e^{-\Delta G(5' \text{ d})/RT} \\ & \times V(i+1,k-1) \times \omega(1,i) + \sum_k W3(k) \times e^{-\Delta G(3' \text{ d})/RT} \times V(i,k-2) \\ & \times \omega(1,k-1) + \sum_k W3(k) \times e^{-\Delta G(\text{TM})/RT} \times V(i+1,k-2) \times \\ & \omega(1,i) \times \omega(1,k-1) + \sum_{k,m} W3(k) \times e^{-\Delta G(\text{CS})/RT} \times V(i,m) \\ & \times V(m+1,k-1) + \sum_{k,m} W5(k) \times e^{-\Delta G(\text{CS})/RT} \times V(i,m) \times V(m+2,k-2) \\ & \times \omega(1,m+1) \times \omega(1,k-1) + \sum_{k,m} W5(k) \times e^{-\Delta G(\text{CS})/RT} \times V(i+1,m-1) \\ & \times V(m+1,k-1) \times \omega(1,i) \times \omega(1,m).\end{aligned}$$

$W3(N+1)$ is initialized to 1.

WC is the energy contribution of coaxial stacking inside a multiloop and is defined by:

$$\begin{aligned}WC(i,j) = {} & \sum_k e^{-\Delta G(\text{CS})/RT} \times V(i,k) \times V(k+1,j) \times e^{-2c/RT} \\ & + \sum_k e^{-\Delta G(\text{CS})/RT} \times V(i+1,k) \times V(k+2,j) \times e^{-2b-2c/RT} \\ & \times \omega(1,i) \times \omega(1,k+1) + \sum_k e^{-\Delta G(\text{CS})/RT} \times V(i,k) \\ & \times V(k+2,j-1) \times e^{-2b-2c/RT} \times \omega(1,k+1) \times \omega(1,j)\end{aligned}$$

$WMBL$, the energy contribution of the last part of a multibranchloop with at least two components is defined by:

$$\begin{aligned}WMBL(i,j) = {} & \sum_k \Big(WL(i,k) - \omega(1,i) \times WL(i+1,k) \times e^{-b/RT} \Big) \\ & + WC(i,k) \times \{WL(k+1,j) + WMBL(k+1,j)\}\end{aligned}$$

WL, the energy contribution of the last part of a multibranchloop with one components is defined by:

$$\begin{aligned}WL(i,j) = {} & V(i,j) \times e^{-c/RT} + e^{-\Delta G(3' \text{ d})/RT} \times V(i,j-1) \times e^{-(b+c)/RT} \times \omega(1,j) \\ & + e^{-\Delta G(5' \text{ d})/RT} \times V(i+1,j) \times e^{-(b+c)/RT} \times \omega(1,i) \\ & + e^{-\Delta G(\text{TM})/RT} \times V(i+1,j-1) \times e^{-(2b+c)/RT} \times \omega(1,i) \times \omega(1,j) \\ & + WL(i+1,j) \times e^{-b/RT} \times \omega(1,i).\end{aligned}$$

Finally, WMB, the energy contribution of multibranch loop with at least 2 components is:

$$WMB(i,j) = WMBL(i,j) + WMB(i,j-1) \times e^{-b/RT} \times \omega(1,j)$$

The probability of a base pair involving nucleotides i and j is:

$$P_{i,j} = \frac{V(i,j) \times V(j,i+N)}{W5(N) \times \omega(0,i) \times \omega(0,j)}$$

This is because $W5(N) = Q$ and $V(i,j) \times V(j,i+N)$ represents the total contribution to Q of secondary structure conformations that contain the base pair of i to j.

Table of sequences, native structures, and structures predicted by RNAsc

Table 1. Sequence, native structure, and structure predicted by RNAsc.

**Sequence / Structure**			
RNA	length	sequence, native structure, and predicted structure	
asp-tRNA*	75	seq.	GCCGUGAUAGUUUAAUGGUCAGAAUGGGCGCUUGUCGCGUGCCAGAUCGGGGUUCAAUUCCCCG<br>UCGCGGCGCCA
		nat.	(((((((..((((........)))).(((((.......))))).....(((((.......)))))<br>)))))))....
		pred.	(((((((..((((........)))).(((((.......))))).....(((((.......)))))<br>)))))))....
HCV IRES	95	seq.	CCAUGAAUCACUCCCCUGUGAGGAACUACUGUCUUCACGCAGAAAGCGUCUAGCCAUGGCGUUA<br>GUAUGAGUGUCGUGCAGCCUCCAGGACCCCC
		nat.	..............((((.((((.....(((((..(((.((...((((((.......)))))).<br>...)).)))..).))))))))))))......
		pred.	..............((((.((((.....((((...(((.((...((((((.......)))))).<br>...)).)))....))))))))))))......
P546	155	seq.	UGCUGAAAUAUCUUCAUUUGAAUAAUAAAUUACUAUAUUAUUCAAUUAAUUAUUUAUAAUAAUA<br>UAAUUUGAAAUAAAAAUAAUAUAGUUAAAAUAUUUAUUAUAAGAAGAAAAUUAGCAGUAAUUAA<br>UAUAUAUAUAUAUAUAAAAUUAAUUAU
		nat.	((((((..(.(((((......(((((((((.(((((((((((.((((((((((.....))))).<br>)))))).........)))))))))))......)))))))))..))))))..))))))((((((((<br>(.(((((....)))))..)))))))))
		pred.	.(((((....(((((......(((((((((.((((((((((((((((((((((.....))))).<br>))))).)).......))))))))))......)))))))))..)))))...))))).((((((((<br>(.(((((....)))))..)))))))))
phe-tRNA	76	seq.	GCGGAUUUAGCUCAGUUGGGAGAGCGCCAGACUGAAGAUCUGGAGGUCCUGUGUUCGAUCCACA<br>GAAUUCGCACCA
		nat.	(((((((..((((........)))).((((.........)))).....(((((.......))))<br>))))))))....
		pred.	(((((((..((((........)))).(((((.......))))).....(((((.......))))<br>))))))))....
5S rRNA	120	seq.	UGCCUGGCGGCCGUAGCGCGGUGGUCCCACCUGACCCCAUGCCGAACUCAGAAGUGAAACGCCG<br>UAGCGCCGAUGGUAGUGUGGGGUCUCCCCAUGCGAGAGUAGGGAACUGCCAGGCAU
		nat.	.(((((((((.....((((((((....(((((((.............))))..)))...)))))<br>).)).((.......((((((((...)))))))).......))...))))))))))..
		pred.	((((((((((((.(.((((((((....(((((((.............))))..)))...)))))<br>).))(((...))).((((((((...))))))))......).))..)))))))))).
glycine	162	seq.	GAUAUGAGGAGAGAUUUCAUUUUAAUGAAACACCGAAGAAGUAAAUCUUUCAGGUAAAAAGGAC<br>UCAUAUUGGACGAACCUCUGGAGAGCUUAUCUAAGAGAUAACACCGAAGGAGCAAAGCUAAUUU<br>UAGCCUAAACUCUCAGGUAAAAGGACGGAGAAAA
		nat.	((((((((......((((((....)))))).(((....(((.....)))...)))........)<br>)))))))........(((((......(((((.....))))).(((....(((....((((....<br>)))).....)))...))).......))))))....
		pred.	((((((((......((((((....)))))).((((((((......)))))..)))........)<br>)))))))........(((((......(((((.....))))).(((...((((...(((((....<br>)))).)...))))..))).......)))))....

References

1. Wilkinson K, Merino E, Weeks K (2005) RNA SHAPE chemistry reveals nonhierarchical interactions dominate equilibrium structural transitions in tRNAAsp transcripts. Journal of the American Chemical Society 127: 4659–4667.

2. Deigan KE, Li TW, Mathews DH, Weeks KM (2009) Accurate SHAPE-directed RNA structure determination. Proc Natl Acad Sci USA 106: 97–102.

3. Duncan C, Weeks K (2008) Shape analysis of long-range interactions reveals extensive and thermodynamically preferred misfolding in a fragile group i intron RNA. Biochemistry 47: 8504–8513.

4. Kladwang W, Vanlang CC, Cordero P, Das R (2011) Understanding the Errors of SHAPE-Directed RNA Structure Modeling. Biochemistry 50: 8049–8056.

5. Mathews DH (2004) Using an RNA secondary structure partition function to determine confidence in base pairs predicted by free energy minimization. RNA (New York, NY) 10: 1178-1190.

6. Mathews D (2005) Predicting a set of minimal free energy RNA secondary structures common to two sequences. Bioinformatics 15: 2246–2253.
